# Using the Transformative Storytelling Technique to Generate Empowering Narratives for Informal Caregivers: Semistructured Interviews, Thematic Analysis, and Method Demonstration

**DOI:** 10.2196/36405

**Published:** 2022-08-02

**Authors:** Milica Petrovic, Silvia Bonanno, Marta Landoni, Chiara Ionio, Mariët Hagedoorn, Andrea Gaggioli

**Affiliations:** 1 ExperienceLab Università Cattolica del Sacro Cuore Milan Italy; 2 Università Cattolica del Sacro Cuore Brescia Italy; 3 Department of Psychology Centro di Ricerca sulle Dinamiche Evolutive ed Educative Università Cattolica del Sacro Cuore Milan Italy; 4 Department of Health Psychology University Medical Center Groningen University of Groningen Groningen Netherlands; 5 Research Center in Communication Psychology Università Cattolica del Sacro Cuore Milan Italy; 6 IRCCS Istituto Auxologico Italiano Milan Italy

**Keywords:** informal care, story, storytelling, stories, patient narrative, digital narrative, informal caregiver, caregiver, caregiving, transformative storytelling technique, audio stories, audio story, digital health, eHealth, empower, fiction, life narrative, audio narrative, self-help, user-centered design, human-centered design, innovation in mental health, mental health, therapy

## Abstract

**Background:**

The transformative storytelling technique is an innovative top-down approach to narrative therapy that aims to provide building blocks for creating flourishing narratives for target groups or populations. This approach acts as a facilitator for implementing the human-centered design in developing digital self-help tools for larger samples or target groups.

**Objective:**

This study applied the transformative storytelling technique, as a new approach in mental health, to develop empowering audio narratives for informal caregivers.

**Methods:**

A narrative inquiry was conducted with 17 informal caregivers (16 women and 1 man) who completed a semistructured interview, “Caregiver Life Story,” acquiring information about the beginning of the role, rising action, and critical point of the role. The participants’ ages ranged from 41 to 84 years, with all participants providing care for at least a 6-month period. This inquiry was guided by the transformative storytelling technique, and aimed to collect data relevant to creating fictional stories based on real-life themes.

**Results:**

Twenty-five overall themes were distinguished across three a priori–set categories, providing narrative building blocks for the informal caregiver life stories. The final empowering caregiver life story was created as an example for this study, demonstrating the application of the transformative storytelling technique in an informal care context.

**Conclusions:**

The creation of empowering stories for populations or target groups in mental health care requires a unified and guided approach that will follow clear guidelines and storytelling principles. The transformative storytelling technique is a first of its kind in the mental health context, representing an initial step in enabling and supporting the creation of meaningful stories and the development of relatable, but productive, narratives. Such narratives have the potential to serve across media and digital platforms for supporting and improving well-being, and potentially triggering self-change in the target group or population.

## Introduction

The narrative approach in therapy is a technique grounded in feminist, anthropological, and multicultural theories, offering a collaborative narrative journey and honoring the life experiences of the client [1]. Following this approach, a narrative therapist assists a client to reauthor the problematic experience into a more constructive personal story by relying on the notion of “multiple truths” within the story rather than the client’s subjective stance.

The therapist works with the “problem-saturated” story the client holds about his/her life or some aspect of life, and collaboratively engages in the process of “unpacking” and “reauthoring” the story. This bottom-up approach aims to mutually arrive at the preferred narrative of the story, deconstructing the experience to emphasize the disregarded but helpful aspects and to reconstruct it into a more positive self-story.

Some of the common applications of the narrative approach can be seen in counseling for depression; recovery from abuse; addiction; posttraumatic reactions; and in therapeutic work with couples, adults, and children [2-4]. However, a significant limitation of the highly personal nature of narrative therapy is the challenge to implement it for a target group or population. More concretely, a target group or population is here defined as a large group of people sharing a common issue (eg, alcohol abuse), difficulty, or mental health problem (eg, posttraumatic stress disorder).

Guided by the idea of expanding narrative therapy principles to the individuals belonging to wider groups and populations, we developed the transformative storytelling technique (TST) [5] as a top-down approach to traditional narrative therapy.

Unlike the bottom-up approach where the clients work on creating a productive self-narrative following various narrative techniques (eg, narration, journaling, mapping the influence of the problem, outcome questions, counterviewing questions), the top-down approach starts from a ready story to be delivered to a specific group (eg, informal caregivers).

In this manner, the ready TST narrative acts as a template and a pathway for assisting with self-story restructure for the individual, with no additional knowledge of narrative techniques needed. Concretely, the TST-structured fictional stories act in a 2D manner. The primary dimension is to assist end users (eg, informal caregivers) to create a more productive self-story within the context (eg, caregiving) by using the fictional TST story as a template. The second dimension facilitates and enables users to anticipate and intercept the potential role-related issues and prepare appropriate coping strategies.

However, outside of the mental health context, the fundamental of storytelling is both art and craft. While art is an instinct-driven process, the crafting part of storytelling requires a technique in the form of a guided approach that builds the story. Some traditional storytelling techniques include Monomyth, also known as the Hero’s Journey; Rags to Riches; The Mountain, or the Freytag pyramid; Nested Loops; Sparklines; In Medias Res; False Start; and Converging Ideas [6]. These techniques provide a cohesive structure for the narrative in literature and allow a writer to set a proper stage for telling the story. For example, the Monomyth, like Joseph Campbell’s Hero Journey, sets the storytelling in a circular plot where the protagonist leaves the known and sets out on a journey [6]. Throughout this journey, the protagonists experience difficulties, despair, and lessons, or encounter teachers that help them move on and return to the starting point as a better and improved version of the self (ie, Hero).

Conversely, the Nested Loops approach enables the core message to be communicated through several narratives delivered within one story. Similarly, the Mountain, which is also known as the Freytag pyramid, allows the storytelling formation in a rising line that marks the dramatic points and resolution of the difficulties [6]. In fact, the Freytag pyramid is one of the oldest and most classic techniques, which is easily utilized and simply structured, allowing even amateur writers to set their story appropriately. Therefore, we adopted this approach in developing our TST.

Unlike the traditional approaches serving the literature structure, the TST provides clear guidelines in creating and structuring empowering digital stories. This technique relies on the narrative approach to therapy and storytelling principles in the narrative, allowing the implementation of narrative therapy principles (eg, narration, storytelling; nonlinear, linear, or chronological narrative formation) to larger groups or members of a target population simultaneously through audio or video stories.

Specifically, with the TST, we aim to address the narrative identity needs (ie, understanding the self in the story) of a group or target population. The TST provides clearly structured digital narratives that act as a cue for triggering self-restructuring within the narrative identity of “the self.” In such a manner, with the TST, we strive to empower end users by giving them a tool that will facilitate the adaptation of adverse or unexpected life circumstances (eg, caregiving) into the productive story of “the self.”

The TST facilitates the process of human-centered design in the creation of digital therapeutic narration for larger groups and populations, and is currently the only existing technique that provides clear guidelines in creating digital stories for mental health. In line with this concept, the TST differs from the traditional storytelling techniques mentioned above because it focuses on structuring the experiences of target groups to produce building blocks for the creation of productive template stories that trigger self-restructure. Moreover, unlike the real-life accounts of individuals, the TST allows integration of multiple experiences within a given context, resulting in richer and more representative storytelling content.

In this study, we focus on informal caregivers as a target population for developing a healing story guided by the TST. Informal caregivers are considered a backbone of the health care systems across Europe, and we define this population as long-term unpaid primary care providers who assist a family member or a close other in need (eg, spouse, parent, cousin, in-law, close neighbor) with daily life activities over a course of an illness or chronic condition.

Informal caregivers of older adults and individuals living with dementia have been reported to experience depressive symptoms, anxiety, and increased levels of stress throughout their role when compared to noncaregivers [7]. Moreover, numerous adverse health outcomes for informal caregivers have been noted across the literature [8-10]. Reflecting on the Italian caregiving context, long-term care provision in Italy as well as other Mediterranean countries has been labeled as “familialist” or family-run [11]. This type of care regime puts the responsibility of providing long-term care for a loved one in need on the family members, who often reach out for additional support mostly from migrant care workers and less frequently from the social and health systems [11].

A closer look at the existing mental health support and interventions for informal caregivers indicates respite care, psychosocial interventions, and information and communication technology support as the most common therapeutic options used by mental health experts [12]. Although numerous studies recognize the existing narrative-identity issues within the caregiving role [13-16], there is no study available that explored the potential of applying or adapting the narrative approach to accommodate shifts in self-identity of informal caregivers. Informal caregivers often place caregiving activities into primary focus within their lives, while personal life and needs become secondary [17]. In this manner, caregivers take the responsibility of providing care for a loved one in need while negotiating newly formed and unfamiliar roles within the relationship and neglecting other aspects of life [17].

In fact, this all-consuming nature of caregiving inevitably interferes with social and relational roles, resulting in identity disruptions [18]. These disruptions mostly occur when personal experiences do not align with the preconceived “self,” ultimately causing distress and loss of meaning in life [18].

Interestingly, informal caregivers report a loss of self as an outcome of the caregiving role, and as the caregiving role becomes more prominent, informal caregivers require meaning-making in the context of their caregiving identity [17,19]. In line with this situation, the ability to reconcile the discrepancy between self-identity and caregiving identity is detrimental in productive adoption of the caregiving role into the narrative identity of self [20].

Accordingly, the aim of this study was to provide a step-by-step demonstration of application of the TST for creating a productive and empowering story in the informal care context. Such a story can then be used as a cue for triggering self-restructuring within the narrative identity of an informal caregiver, and serve as a guide to facilitate familiarization and understanding of the caregiving experience for the caregivers early in the role.

## Methods

### Overview of the TST

**Figure 1:**
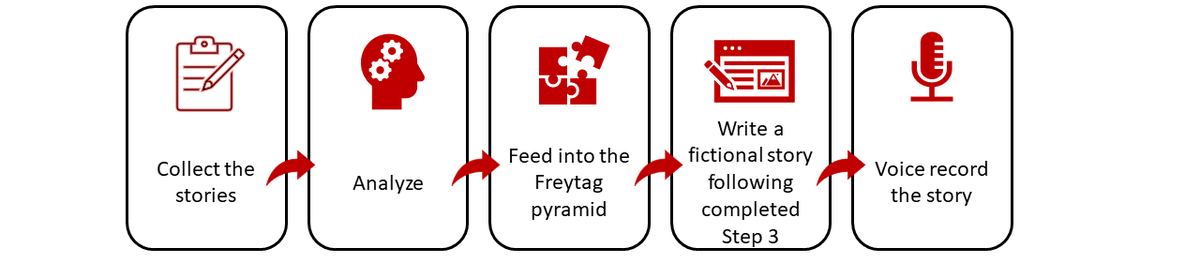
Steps of the transformative storytelling technique.

The TST is a top-down approach to traditional narrative therapy that we developed to create ready stories with flourishing narratives for larger groups or target populations. In this study, we used the TST for the creation of audio stories, built through a series of five consecutive steps (see Figure 1), acting as a concrete guideline that we follow in developing the empowering digital story for an informal caregiver sample.

The therapeutic aspect of the TST is based on narrative principles in therapy and draws from the theory of narrative identity [21], which poses the identity of “the self” as a construct based on an internalized and evolving story required for the meaning-making about the self and others. The storytelling aspect of the TST is based on the Freytag pyramid, which was introduced in 1894 for developing engaging and dramatic story plots in literature [22]. This pyramid is a mountain type of storytelling technique, commonly used in narratives owing to its simplicity, which allows use by individuals who are not experienced writers.

By applying the TST, we (1) collected the stories of the target population (ie, informal caregivers) through structured interviews; (2) thematically analyzed the stories in search for themes; (3) fed the obtained themes into the Freytag pyramid structure, which enabled visualization of the key elements for (4) writing the fictional story that will be (5) audio-recorded with voice actors, and used as a blueprint of a flourishing narrative and a cue for triggering self-restructuring within the narrative identity of the target population. The fictional story is freely constructed, while strictly following the building blocks retrieved in step 3. Therefore, the obtained story is just a sample of numerous potential fictional stories that can be created following the step-3 building blocks. This paper complies with Standards for Reporting Qualitative Research, as recommended by O’Brien and colleagues [23].

### Ethical Statement

The study was approved by the Commissione Etica della Ricerca in Psicologia (CERPS) Ethical Committee of Università Cattolica del Sacro Cuore (UCSC) under protocol number 20-21, and was conducted in compliance with the latest version of the Declaration of Helsinki. Informed consent, provided to the participants in a written version, included a page containing an information sheet about the study and a page addressing the General Data Protection Regulation, as well as the participant’s right to withdraw from the study at any point with no penalties. The informed consent followed the template provided by the CERPS of UCSC and was additionally adapted to include the specific information about the study.

### Participants and Procedure

The participants were long-term primary caregivers (eg, spouse, daughter, daughter-in-law) of an elderly family member or close other who requires care due to a chronic condition, advanced age, or age-related illness (N=17; 16 women, 1 man). They were recruited on a voluntary basis through a nonprofit caregiver association in Italy. The participants had to be providing primary care at the point of recruitment for at least a 6-month period regardless of the living conditions (living with the care recipient or living outside of the care recipient’s home). Several recruitment strategies were employed, including posting an information sheet about the study on the information corner in the association, telephone calls to the members of the association who indicated they would like to take part in future studies, and through announcements by the self-help group facilitators who disseminated the study and referred interested caregivers to the information corner where the information sheet was available.

Participants received a link for the online interview through email or during their visit to the caregiving association by the group facilitator or researcher (SB), depending on their presence within the association. The structured interview was available via the Type Form platform, with an average completion time of 10 minutes. Some of the participants who expressed the desire to participate in the study but did not feel comfortable due to the ongoing pandemic or were not sure of how to use the Type Form website were assisted by the researcher in charge of data collection (SB). Furthermore, due to COVID-19 restrictions at the time of data collection, most caregivers chose to be contacted via telephone to prevent potential contagion of virus transmission to the care recipient. The researcher performed the interview and filled in the data in Type Form on behalf of the participant. The telephone interviews were audio-recorded (at the researcher’s request and upon participants approval) and then transcribed into Type Form by the researcher. All participants agreed to be recorded. The duration of the structured interview varied (10-30 minutes) depending on the amount of information shared by the participants, and each participant was interviewed only once.

### Interviews

A structured interview, named “Caregiver Life Story,” was created for the purpose of this research. Following the general form suggested by the TST, the interview questions inquired about (1) the beginning of the caregiving role/issue/event, (2) changes that took place, (3) the new routine/outcome, (4) psychological and emotional challenges of the caregiving role/issue/event, and a (5) a critical/climax point of the issue/role/event.

In addition, the interview assessed demographic (ie, age, gender) and care recipient illness-related (ie, age and health condition of the care recipient, relationship with the care recipient) information.

### Analysis

**Figure 2:**
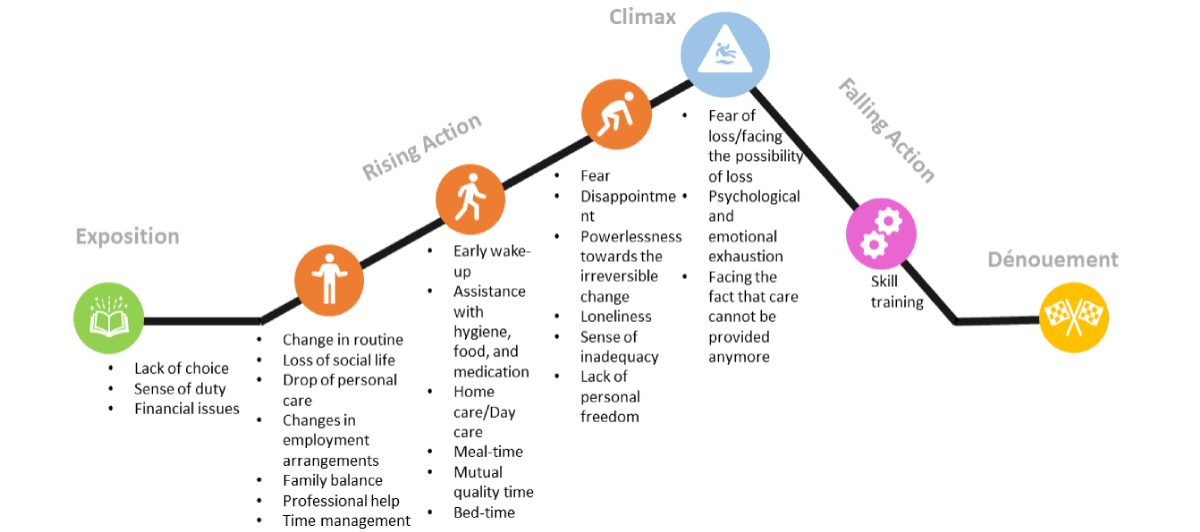
Themes inserted into the Freytag pyramid serving as building blocks for a story.

Thematic analysis was performed following an inductive approach and the six-phase guidelines proposed by Braun and Clarke [24]. The phases included (1) familiarization with the text, (2) initial coding, (3) search for themes, (4) revision of themes, (5) definition of themes, and (6) final output (see Multimedia Appendix 1). Within the TST, each question acts as an a priori category derived from the Freytag pyramid. In essence, each category collects the data for a specific part of the story plot (deductive) of the Freytag pyramid (see Figure 2). To ensure the extraction of relevant data, we performed the initial coding, which was then refined with more specific and narrowed codes that allowed for an easier search for themes and definition of themes. The data were analyzed by two researchers independently. Namely, a large amount of interview data contained particularly emotional responses and confessions from the everyday life of informal caregivers that could potentially influence the interpretation. To mitigate the potential effects that highly personal and emotional recollections shared by the participants could have on the objectivity of the analysis, independent analyses were performed by two researchers (MP and ML) and the findings were then jointly compared and discussed until potential disagreements were resolved. The final codes and themes were established by noting and sorting the overlapping codes from the researchers’ analysis. The third researcher (GA) supervised this process of distinguishing the themes independently and establishing the final themes jointly. In instances where the researchers disagreed, the points of disagreement within data were marked and blindly validated by the third researcher (GA) without prior knowledge on the specifics of the disagreement.

## Results

### Participants

The sample consisted of 16 female informal caregivers and one male informal caregiver (see Table 1). This number generally reflects the unequal gender distribution in informal care, pointing to women as the usual primary caregivers [25]. The most common illness among care recipients was Alzheimer disease, often associated with other chronic conditions. Interestingly, the gender distribution among care recipients was quite homogenous in contrast to the caregivers being predominantly women. Participants provided care mostly for a parent or both parents at once, while there were also some participants providing care for a mother-in-law.

The following results are presented in line with the steps of the TST (see Figure 1 and Multimedia Appendix 1 for the detailed coding procedure).

**Table 1:** Characteristics of the study population.^a^

Participant ID	Gender	Age (years)	Gender of the care recipient	Age of the care recipient (years)	Relationship to the care recipient
P1	Female	41	Male	70	Father
P2	Female	62	Male	67	Husband
P3	Female	46	Female	82	Husband’s aunt
P4	Female	55	Female	77	Mother-in-law
P5	Female	57	Female	80	Mother-in-law
P6	Female	61	Female	NA^b^	Mother
P7	Female	78	Male	79	Husband
P8	Female	55	Male	81	Father
P9	Female	63	Male	68	Husband
P10	Female	73	Male	78	Husband
P11	Female	47	Male and female	80 and 80	Parents
P12	Female	64	Female	85	Mother
P13	Male	84	Female	NA	Wife
P14	Female	65	Male and female	91 and NA	Parents
P15	Female	74	Male	80	Husband
P16	Female	57	Female	84	Mother
P17	Female	59	Female	82	Mother

^a^Illness-related data are removed to respect the privacy of the participants.

^b^NA: not available; information not shared or recipient recently deceased.

### Step 2: Analysis of the Stories

#### Overview of Themes

The thematic analysis resulted in 25 overall themes. A full list of initial coding, refined coding, and theme formation within each category is available in Multimedia Appendix 1. The 25 final themes were sorted into a priori categories of the TST. Each interview question collected data for a specific category: (1) the beginning, belonging to the category of *exposition/the beginning*; (2) life changes, daily life, and psychological and emotional challenges, belonging to the category *rising action*; and (3) critical point, belonging to the category *climax/critical point*.

#### Exposition/The Beginning Category

The themes retrieved in the first category reflect the role onset/exposition, which include *the lack of choice*, *sense of duty*, and *financial issues*. The role onset has been marked with a *lack of choice* in accepting the caregiving duty either due to the living condition (eg, spouse), or the lack of another caregiver available or willing to provide care (eg, other siblings denied caregiving, children living across the country), often imposing the caregiving role upon the only available family member.

The *sense of duty* in providing care was the second noted theme. The sense of duty appeared due to a personal understanding of the relationship, family ties, marital responsibility, or being the only child that is willing to assume care, hence feeling as the last resort available, which is in essence closely related to the first theme of a lack of choice. The caregivers described the sense of duty with statements such as “I have to, she is my mother”; “I am the wife”; or “I am the daughter it’s my duty.”

The third theme of the first category, *financial issues*, appeared at the beginning of the role. In line with the theme lack of choice, caregivers also reported that the financial aspect has been an important factor in their decision to become a caregiver. There was a noted lack of financial resources for care homes as well as in-home formal caregiver support, which also seemed to further add to the prolonged care even when the caregiver was not willing to continue the care provision.

#### Rising Action Category

##### Main Category

This category explores the rising action through reported daily changes that occur in individuals’ lives due to the caregiving role, average daily life after the changes occurred, and finally psychological and emotional challenges of the role. The category *rising action* constitutes the following three subcategories.

##### Daily Changes Subcategory

Seven themes were noted within the daily changes subcategory. Caregivers reported that a *change in routine* took place after the role was assumed, which forced them into giving up numerous habits and adjust to the new way of living. The caregivers expressed this through statements such as “I lost my routine,” “the day was organized in the service of her,” “I gave up my personal time,” and “had to be more present for my parents.”

The following theme within the subcategory daily changes was the *loss of social life*. Caregivers noted this as a relevant aspect of the daily changes that took place through shared statements such as “loss of friends”; “lack of social life”; “gave up my social life”; “all activities we had together stopped, hence our friends drifted apart”; and “my social life doesn’t exist anymore.”

The third theme, the *drop of personal care*, was evident through the lack of sleep or sleep disruption, limited sleeping hours, strict waking-up hours, loss of freedom for personal time and activities with family and friends, as well as lack of time to visit the doctor for personal health reasons. This theme was reflected in statements such as “I cannot take care of my own health issue, nor make the time to visit the doctor”; “I have to monitor her during the night because she already fell once”; and “my mother remains my priority.”

The fourth theme of the daily changes subcategory was *changes in employment arrangements*, which appeared in all cases where employment existed prior to the caregiving role. Some of the statements supporting this theme included “full-time to part-time work,” “I had to quit my job,” “I was waking up at 4:30 AM going to work and returning home at 8:30 AM.”

The fifth theme in the subcategory daily changes was *family balance* disruptions, which reflects internal family problems that informal caregivers experienced in different contexts due to their role. Disrupted family time or activities were reported among spouses, parents and children, and siblings. The disruptions involved changes in the usual weekend-routine patterns of a family, the amount of time spent together, vacation routine, free time among parents and children, and free time for spouses. Moreover, the family balance was also affected among spousal caregivers reporting that what used to be regular mutual activities have now ended. Additionally, the family balance has also been affected over a course of caregiving due to lack of involvement of other family members who were considered obliged to caretake (eg, brothers not assisting with caregiving to sister, daughters not assisting with caregiving activities to mother), resulting in the deterioration of the overall family relationship.

The sixth theme, *professional help*, was noted when caregivers addressed the professional help either in the form of day care or professional/semiprofessional caregivers who were hired for in-home assistance. The caregivers who had the financial resources to cover the in-home professional/semiprofessional care expenses hired help regardless of the living arrangements or their full-time presence in the home. Some of the statements the caregivers made regarding professional help included “I paid the woman to help me,” “I had to send him to day care against his will so that I can work,” and “I had 24-hour home assistance.”

The seventh theme of *time management* within the rising action subcategory of daily changes included all the relevant issues regarding the daily organization of time and changes that took place within the personal schedule. For instance, caregivers reported “all day revolved around the care recipient,” “the day was organized in the service of the care recipient,” and “I had to give up my personal time.”

##### Average Day Subcategory

The average day subcategory explores a day in the life of an informal caregiver, including all of the relevant activities concerning care after the initial routine has been shifted and caregiving became a part of life. We noted six emerging themes within this subcategory pointing to *early waking hours*; *assistance with hygiene, food, and medication*; *daycare center/home*; *meal time*; *mutual quality time*; and *bed time*.

Informal caregivers reported *early waking hours* as the beginning of each day, followed by *assistance with hygiene, food, and medication*. These two themes were reported by all caregivers regardless of the living arrangements (eg, living together or separately). The caregivers reported that the first activity in the morning involved assistance with showering, cleaning, dressing, breakfast, medications, and therapy. This assistance was provided alone or, in a few cases, together with the professional/semiprofessional in-home caregiver. Caregivers also noted that the assistance with personal hygiene was the most uncomfortable aspect of the caregiving role and the aspect that was also seen as repulsive in the beginning of the caregiving role.

The third theme was *home care/day care*. Care recipients either visited the daycare center for a couple of hours, or home care continued with the existing daily routine such as brief walks, grocery shopping, and reading newspapers. Caregivers who had the opportunity to spend some time away from the care recipient noted that they felt good about these hours even though they were still occupied with the caring obligations.

The *meal time* theme followed the home care/day care theme. The care recipient would usually return from the daycare center or the late-afternoon home care would continue with early dinner. The *mutual quality time* theme was noted as a small period existing after dinner where the care recipient and caregiver spent quality time together, such as listening to music, talking, reading, or watching TV. These moments were also marked with the occasional efforts of informal caregivers to reminiscence with the care recipient about the past and good moments lived together.

*Bed time* was the final theme within the average daily life subcategory, in which caregivers prepared the care recipient for sleep, distributed the medication, assisted with other care needs, and helped the care recipient go to bed. The caregivers mostly reported going to bed sometime later, after small house chores were complete.

##### Psychological and Emotional Challenges Subcategory

Psychological and emotional challenges of the role were identified through six emerging themes, including *fear, disappointment, powerlessness, loneliness, sense of inadequacy*, and *lack of personal freedom*.

The theme *fear* was noted as an important aspect of the subcategory emotional and psychological challenges. Fear experienced by caregivers was related to the care recipients’ health and possible deteriorations of their health. In instances where the care recipient was ill, or the condition suddenly worsened, the caregivers reported fearing the possible outcomes and/or that they will not be able to appropriately assess the seriousness of the condition, which might have severe or lethal consequences on the care recipient.

Following the theme of fear, the *disappointment* theme emerged in the subcategory of emotional and psychological challenges as caregivers described daily events that affected them the most. It was noted that caregivers were disappointed with the disease progression and the outcomes, stating that related events are particularly difficult since they are taking place right in front of the caregivers. Some of the statements portraying these themes include “seeing your mother not recognizing you and not knowing anymore,” “becoming a mother of my parents,” and “I have always seen my parents as pillars and instead I discovered all their weaknesses.”

The *powerlessness* theme appeared as an outcome of the irreversible change in the disease progression, when, despite the care provided, the condition was still deteriorating/not improving. Caregivers reported “the inability to do something against the disease/the sense of powerlessness caused by the disease weighted the most,” “maintain a normal relationship when you are overwhelmed by the suffering that involves seeing the decline due to the disease,” and “I feel so angry and I feel lonely.”

*Loneliness* was noted as a fourth theme of the psychological and emotional challenges subcategory, overlapping with the loss of social life theme noted in the subcategory of daily changes. Loneliness was identified by statements such as “I feel lonely,” “I find it hard to be the only one who has to follow him,” and “she is refusing other caregivers’ presence.”

*Sense of inadequacy* was the fifth theme noted when the care recipient condition worsened or difficult symptoms occurred. Caregivers reported feeling as if they were not sufficiently or adequately prepared to manage caregiving duties, or being in need of time to adapt to new circumstances/worsening of the condition. The examples were noted through a variety of statements such as “the moments of aggression that he had”; “feeling inadequately equipped to provide assistance”; “my movement had to be exactly the same as hers and this cannot happen overnight, you have to adapt”; “she once grabbed my neck”; and “she was waking up at night and ran.”

Finally, *lack of personal freedom* was the sixth theme caregivers repeatedly noted in the subcategory of psychological and emotional challenges. This theme was reflected in planning out private time, going out alone, socializing, organizing weekends with family, or simply managing to spend time with people other than the care recipient. Furthermore, caregivers reported a lack of personal freedom in statements such as “lack of freedom is what oppresses me,” “inability to spend time with children,” and “sense of guilt for not having the time for everyone.”

#### Climax/Critical Point Category

The climax point in the TST is the peak moment where the story begins to unravel since it reached the moment of the greatest tension. The climax/critical point category reflects the peak point for informal caregivers, where the provision of care has reached its peak in terms of difficulty, burden, and negative experiences, and hence must change or terminate. This category explores the themes emerging from the experiences of the most critical moments in the role of caregiving.

The initial theme recognized within the critical point category was the *fear of loss/facing the possibility of loss*. Caregivers reported that the critical points occurred when they were confronted with the further/serious health deterioration of the care recipient that implied the possibility of a lethal outcome. This theme appeared in the later phases of caregiving and could be potentially related to the following theme of *psychological and emotional exhaustion*. Concretely, when the caregiver had the resilience and strength to carry the role-related burden, the fear was less present/obvious. Some of the caregivers’ statements shedding light on the theme of fear of loss/facing the possibility of loss include “when he gets ill/does not feel well I am scared because I believe it is serious”; “while my father was still having a driving license, since he was a danger to himself and others”; “when he is ill I do not know what the outcome will be”; and “the most critical moment is the last days when you know that nothing else could be done.” This fear appeared to surface after the first encounter with the critical situation where the life of a care recipient was jeopardized.

The theme *psychological and emotional exhaustion* was reflected through numerous examples of negative emotions experienced, tiredness within the role, and the concern about the continuation of the caregiving activity. Furthermore, a few caregivers reported that due to psychological and physical fatigue—which could, in turn, be the consequence of psychological exhaustion—they were concerned if caregiving could last any longer. Caregivers expressed this through statements such as “I have experienced all the feelings of this world, anger, frustration, resentment, loneliness”; “I feel alone and criticized by my siblings while they are not truly aware of the situation”; “yes, there are mornings when I’m more tired and hearing the same things over and over again is heavy”; and “physical fatigue that prevented me from seeing the situation clearly/the worst moment was when I realized I cannot bear the caregiving for much longer.”

The final theme noted within the category of climax/critical points was *facing the fact that care cannot be provided anymore*. It seems that such confrontation with the reality of the situation comes after first experiencing physical, psychological, and emotional exhaustion. Such a conclusion could also be related to the previous argument about the themes of fear, disappointment, and powerlessness, argued as early indicators that imply the caregiver is reaching a critical point within the role.

### Step 3: Arranging Themes Into the Freytag Pyramid

The obtained themes were used as building blocks for development of a fictional story guided by the real-life experiences common to the target population (ie, informal caregivers). The real-life experiences are reflected in the obtained themes, while the TST is used for incorporating these real-life experiences into a flourishing narrative. Following the third step of the TST, the obtained building blocks/themes were fed into the Freytag pyramid (Figure 2) and used for the creation of numerous stories and different narratives for informal caregivers.

### Step 4: Creation of the Final Story

The fourth step of the TST consists of writing a fictional story using the obtained figure from step 3 (see Figure 2). The created fictional story provides the viewer/listener an identification with the protagonist who experiences similar challenges throughout the role (ie, identification is supported by the building blocks/themes) but also an emotional distance that allows taking an objective stance toward the caregiving experience through a third-person perspective (ie, the narrator telling the story to the caregiver, or the protagonist). A sample of a fictional story created as step 4 of the TST is included in Textbox 1 (see Multimedia Appendix 2 for the full story).

Once the final step of the TST is completed and the written story is audio-recorded using a voice actor, the audio will be available on the Voice Me Out platform we designed for the informal caregivers (the platform is not yet publicly available).

**Final fictional caregiver stories created. Words in italics are the retrieved themes incorporated into the fictional story.**

- Excerpt from the category “The beginning”

I think I can describe it simply; I *did not have a choice*, but to take her with me that night. *“It is my duty”*—I said to myself—because she is my family. Besides, I *cannot afford anyone* to take care of her. As the days were going by, I had to change so many things in my life, one after another. I had never expected all of it to happen that way. I had to reorganize the space in my house and empty a room for her.

- Excerpt from the category “Rising action”

My entire morning routine had to adapt to her from day one. I had to *change my working hours* and shift to *part-time work* because I couldn’t get to work by 9 any longer and stay there until 5. It is difficult sometimes to follow my own schedule.

My spouse often complains, and I do understand him, but I don’t see what else I can do. I can’t put her into a nursing home. My children didn’t seem bothered at the beginning. In fact, they were excited to have Grandma home, but there comes a time when they try to make plans for us to go to the park on the weekend, as we used to, but I can’t promise anything because I do not know if she will be in her good mood that weekend and able to leave the house. My *family balance* is completely disturbed.

- Excerpt from the category “Climax/critical point”

*Fear*, this is how I can describe such moments. I did not know what to do. I was scared for her, for my children. It was fear that didn’t let me think for a moment. I felt *powerless* because she wasn’t getting any better; she was either the same or getting worse. I couldn’t make her get better and I think I was making her worse by not knowing what to do exactly. I was *completely alone*. No one could truly understand what I was going through. I didn’t know what to do and I had no one to call. Everyone I knew had already advised me many times to consider a nursing home. I couldn’t just take a break to have a *moment for myself* to think. It had been like that for months. I had lost my *personal freedom*. I felt trapped.

I took the phone and called an ambulance.

## Discussion

### Principal Findings

This study implemented the TST [5] for developing empowering narratives in an informal care context. We demonstrated how to use the TST to obtain and categorize building blocks of a universal informal caregiver life story to develop an empowering fictional story. The final 25 themes have been distinguished across three a priori categories (ie, the beginning/exposition, rising action, critical point), covering a wide range of experiential, practical, and emotional changes, along with experiences throughout the role.

Numerous obtained themes (eg, within the beginning, and psychological and emotional challenges categories/subcategories) are in line with the past literature and reflect the important aspects of the informal care experience. Although some of the themes such as those within the subcategory of daily life constitute a major part of informal caregiver life and activities, they have been rather neglected in the literature to date (see Figure 2). It can be suggested that some of these themes also have an important role in the caregiver burden.

Interestingly, following the TST, we obtained qualitative data that have already been reported in the literature, but never gathered in one study. Namely, the TST enabled an all-encompassing approach to collecting, structuring, and grouping these findings chronologically. In other words, the TST enabled and facilitated gaining a magnified overview of the detailed caregiving experience.

Findings in the beginning/exposition category, which reflects on the early experiences in the role, indicate that informal care is often taken because of personal beliefs about family duties and obligations. This theme is also in line with prior research noting that informal caregivers consistently report this sense of duty across different cultures [26-28]. Additionally, when several children were available to provide care for a parent, other siblings commonly expected the female child or the oldest child to assume the caregiving role.

Therefore, the sense of duty is arguably not equally present in all potential caregivers, but rather formed by certain expectations for the specific family member to assume care (eg, the female sibling or the oldest sibling). It could be suggested that the sense of duty or the internal perception of duty is one of the distinctions between those that will assume care in the future and those that will refuse it. However, no current data are available comparing the sense of duty among siblings when more than one child is available to assume the role. Furthermore, it can be added that the existing perception of the feminine nurturing role additionally disadvantages female family members when the caregiving arrangements are being determined [29].

The financial aspect/lack of resources for formal or institutional care appeared as an important theme in the role assumption. Informal caregivers that had resources to support formal care or financially cover the placement into the nursing home/daycare center reported such actions early in the role. Moreover, caregivers who were able to afford assistance of any kind reported fewer caregiving tasks, ultimately allowing more personal freedom and time for other activities.

In line with this argument, findings from a recent study across 12 European countries assessing existing care policies and health of informal caregivers point to a significantly lower health status of informal caregivers who provide home-based care [30]. Similarly, our results indicate that in instances where informal caregivers were not able to afford any type of help, the emotional experiences in the subcategory of psychological and emotional challenge within the rising action category were described to a greater extent, with vivid examples and recollections of numerous difficult situations. Another interesting finding is that caregivers who provide only home care reported more severe emotional responses toward the care recipient and frustration toward the caring situation, which was reflected in the rising action category exploring the psychological and emotional challenges concretely. This insight can also be related to the past literature pointing to psychologically and physically abusive behaviors occurring in the informal caregiving context [31-33], suggesting that the type of care (in-home only vs shared care with respite/or a day center) could be related to more severe reactions.

Informal caregivers noted the loss of social life and personal time early in the role, describing these experiences as part of the changes that took place due to the role. A similar theme reemerged as loneliness in the category of psychological and emotional challenges, described mostly as “lack of friends,” “lack of support group,” and “lack of people that can serve as a reference point or companion during the difficulties.” Interestingly, these findings are consistent with a previous study noting the presence of similar experiences of social isolation and lack of support [34].

The relevance of the theme *drop of personal care* can also be aligned with the existing literature pointing to the gradual decrease in the physical health of informal caregivers who provide care for a longer period [35]. Arguably, the drop of personal care can be attributed to the maladaptation to two other themes, *loss of social life* and *changes in the routine*, ultimately occurring as an unconscious process in the caregiver. Moreover, a drop in personal care may act as a potential mediating factor in the decrease of physical health noted in the literature. The past literature exploring personal self-care in informal caregivers underlines the strong association between self-care and emotional well-being, pain, perceived stress, and general health [36].

Changes in employment arrangements is another theme in line with numerous studies, pointing either to the lack of social policy that will provide flexible working hours for informal caregivers or inadequate long-term financial support for the hours missed from work (see [30,37,38]).

Caregiver fear, discovered as another theme, has also been noted in the literature in the context of specific illnesses such as pulmonary fibrosis, amyotrophic lateral sclerosis, and heart failure (see [39-41]), where caregivers did not know how to manage specific illness-related aspects of care. However, the role of an overall fear in informal caregivers about assuming and providing caregiving has been rather neglected.

Another interesting theme discovered in this study is the sense of powerlessness. This theme emerged right before the critical/climax point category. In fact, the themes of fear, disappointment, and powerlessness toward irreversible change could be argued as early indicators that the caregiver is reaching a critical point in adverse experiences within the role, where the care provision either needs to be terminated, restructured, or shared to avoid critical situations.

Sense of loneliness and social abandonment/isolation are present from the early stages of taking up the role and seemed to be most strongly expressed in the emotional and psychological challenges subcategory. These themes are also consistent with the literature, pointing to the reported sense of isolation and abandonment in caregivers (see [42-44]).

The aforementioned themes within the psychological and emotional challenges subcategory could be argued as a set of early indicators leading to the confrontation with the fact that care can no longer be provided, as a part of the critical point category. Following such a perspective, if the early indicators are not appropriately addressed, the critical point follows or can be avoided by perpetual therapeutic work on fear, disappointment, and powerlessness within the role.

Following the categorization of the building blocks (Figure 2) and the creation of the empowering informal caregiver fictional story, these findings demonstrate how the TST can act on two levels. First, the categorization of building blocks with the TST can serve as an informative pathway for further research and interventions. Concretely, building blocks provide a path of the informal caregiver life experience from the beginning of the role until the critical point, and as such, proper steps can be developed to prevent, intercept, or manage problematic experiences in practice.

Second, the value of the created story and the potential effects of this story on informal caregiver well-being need to be assessed in a pilot trial, ensuring that informal caregivers benefit from such stories. The stories serve as a sample of a productive caregiving narrative to be used as a template in self-restructuring of the personal caregiving life stories. If TST stories show positive outcomes for informal caregivers in the currently ongoing pilot testing, we can further explore the development of flourishing gender-adapted narratives through digital health tools. This approach would further facilitate human-centered design in eHealth tools for informal caregivers, and provide a new method of exploring adverse experiences while offering a path for creating flourishing and empowering digital narratives.

### Limitations

Unlike the traditional storytelling approaches, the TST allows for the collection of universal building blocks for target groups or populations rather than individuals. In this sense, the technique enables in-depth exploration of the dominant/repetitive themes in the concrete categories of experience, acting as building blocks of an individual narrative within the target population.

Such collection and representation of the building blocks provide (1) an informative template for research and practice, (2) a pathway in the design and development of tools and interventions, (3) an overview of the narrative of the experience, (4) a blueprint for self-restructuring within the personal story of “the self,” and (5) a top-down approach to narrative therapy (ie, providing ready empowering narratives linked to personal experience).

Several limitations of this study must be considered. Further work on gathering and comparing themes in informal care following the TST is required to verify the universality of the retrieved themes in acting as building blocks for informal care narratives. Therefore, although our focus was on distinguishing universal caregiving experiences through the TST, we cannot assume the generalizability of our findings until the ongoing pilot trial data are retrieved and until the retrieved themes are reproduced by using the TST in further studies. The majority of our sample was predominantly female informal caregivers, leading to possible perspective bias. The male experiences need to be collected and compared against the obtained themes to ensure the generalization of themes as building blocks for informal care narratives. In this sense, minimizing bias in future studies would strengthen the scientific rigor for obtaining universal themes of the informal care experience. Another consideration regarding the scientific rigor that must be acknowledged is that all the participants had already taken part in self-help group meetings of the association, which might have prepared them and facilitated their capacity for self-reflection but likely also contributed to preconception of the emotional difficulties. In this sense, some experiences shared with our researcher (SB) might have already been predefined and conceived in self-help groups rather than reflected on for the purpose of our interview. This might ultimately affect the validity of the reported experiences, since they might have been reassessed and adapted in a self-help meeting to better resonate with the support group members rather than resonating with the general informal care experience.

Moreover, it must be noted that this study did not define an upper age limit for informal caregivers, allowing even elderly caregivers to take part in the study. The age difference among informal caregivers raises a question about the universality of the experiences across generations.

Finally, our sample consisted of informal caregivers who provided care for family members with concrete illnesses such as Alzheimer disease, Parkinson disease, or dementia. Therefore, the obtained experiences in this study could be potentially biased or formed by the specific illness presented in the care recipient. A larger sample of informal caregivers including other chronic conditions would be desirable in better exploring and creating universal themes of the informal caregiving experience.

### Conclusions

The rapid expansion of storytelling applications in mental health calls for structured and tested techniques that will provide consistent narrative structures with clearly defined purpose and goals. The TST is the result of our effort to create a technique for developing structured, meaningful, and empowering narratives for larger groups or populations that can be easily adapted and applied to a digital format. The translation of personal narratives into empowering digital stories is one of our next goals in implementing the human-centered design in digital health development for informal caregivers, where the value of every individual is recognized in the overall process of the group experience.

## Appendix

Multimedia Appendix 1 Phases of theme development.

Multimedia Appendix 2 Final story (translated to English).

## Abbreviations

CERPS: Commissione Etica della Ricerca in Psicologia
TST: transformative storytelling technique
UCSC: Università Cattolica del Sacro Cuore
