# Correction: Using the Transformative Storytelling Technique to Generate Empowering Narratives for Informal Caregivers: Semistructured Interviews, Thematic Analysis, and Method Demonstration

**DOI:** 10.2196/42323

**Published:** 2022-09-14

**Authors:** Milica Petrovic, Silvia Bonanno, Marta Landoni, Chiara Ionio, Mariët Hagedoorn, Andrea Gaggioli

**Affiliations:** 1 ExperienceLab Università Cattolica del Sacro Cuore Milan Italy; 2 Università Cattolica del Sacro Cuore Brescia Italy; 3 Department of Psychology, Centro di Ricerca sulle Dinamiche Evolutive ed Educative, Università Cattolica del Sacro Cuore Milan Italy; 4 Department of Health Psychology, University Medical Center Groningen, University of Groningen Groningen Netherlands; 5 Research Center in Communication Psychology, Università Cattolica del Sacro Cuore Milan Italy; 6 IRCCS, Istituto Auxologico Italiano Milan Italy

In “Using the Transformative Storytelling Technique to Generate Empowering Narratives for Informal Caregivers: Semistructured Interviews, Thematic Analysis, and Method Demonstration” (JMIR Form Res 2022;6(8):e36405), one error was noted.

In the originally published paper, Affiliation 6 for author Andrea Gaggioli appeared as follows:

Applied Technology for Neuro-Psychology Lab, Istituto Auxologico Italiano, Milan, Italy

In the corrected version of the paper, Affiliation 6 has been revised as follows:

IRCCS, Istituto Auxologico Italiano, Milan, Italy

The correction will appear in the online version of the paper on the JMIR Publications website on September 14, 2022, together with the publication of this correction notice. Because this was made after submission to PubMed, PubMed Central, and other full-text repositories, the corrected article has also been resubmitted to those repositories.

