# Supplementary material for: Using the Transformative Storytelling Technique to Generate Empowering Narratives for Informal Caregivers: Semistructured Interviews, Thematic Analysis, and Method Demonstration
Source: JMIR Form Res. 2022 Aug 2;6(8):e36405. doi: 10.2196/36405 (PMC9382549; doi:10.2196/36405)
Supplement: Multimedia Appendix 1 [file formative_v6i8e36405_app1.docx]

| Category  (Questions) | Initial codes - Quotes | Refined codes | Final themes (e.g., refined codes used) |
| --- | --- | --- | --- |
| Exposition  (5. Can you describe how did you become a caregiver, which event led to this situation?) | Passing of a parent;  Worsening of the health; family reasons; the only choice available; sudden health deterioration; no other home available; could not afford care; other siblings not involved; living arrangements; availability; ability to manage illness; sharing the household for 20 years; caregiving always present since marriage; the destiny; love and duty; able to manage illness; I am the daughter; he is my husband; children not available to provide care; refuses external assistance; cannot afford external assistance; they are my parents; cannot afford caregiver; siblings refused to help; illness; after surgical intervention; the only sibling that accepted to be a caregiver; only sibling available; out of duty | No other choices available; obligations due to family ties; financial resources not available for paid care; involuntary acceptance upon the health issue appeared; living arrangements-imposed care; family duty. | **Lack of choice** (e.g., no other choice available, involuntary acceptance upon health issues appeared, imposed care);  **Sense of duty** (e.g., obligations due to family ties, family duty);  Financial issues (e.g., financial resources not available for paid care). |
| Category  (Question) | Initial codes - Quotes | Refined codes | **Final themes** (e.g., refined codes used) |
| Rising action  (6. What are some of the changes that you had to make in your everyday life in order to incorporate the caregiving role?) | From full to part-time job; in-house paid help; hygiene care for the patient; apply changes in routine; day care center; drop social interactions; drop personal routine; family time; work time; lower the social life; everything revolving around care recipient; usual daily activities; change family balance; lose freedom; day care center; organize the day in the service of the care recipient; family involved in all changes; help with hygiene of the care recipient; had to give up everything; daughter cannot help; find help in the house; giving up personal time; family vacations with her; husband neglected; arrange shopping and medical appointments; take a long leave from work; left job; daughters moved far away; lost privacy, friends drifted apart; lost social life; I can never leave him alone; no time for anything; social life does not exist; no sleep at night; cannot care for personal health; relationship with daughters worsened; no social life; home stay; adapt working hours; gave up habits; quit job; family balance; give up self; cannot go home for longer time; monitor her during the night; left job; limited freedom; give up traveling; give up personal things; hire caregiver; return to minimal work hours; no vacations with husband; give up time with daughters; work part-time. | Shifts in routine;  Changes in usual/regular patterns of living; Adopting new responsibilities; Drop of social activities/loss of social activities/time constraints for social activities; changes/disturbances in the usual family functioning; limited/non existent time for personal needs; formal help hiring/full-time help in the house; work pattern shift/full-part time/job loss; time constraints/time-giving up/lack of time for personal things; family time limits/family patterns change/limited family interaction. | **Change in routine** (e.g., shifts in routine, adopting new responsibilities);  **Loss of social life** (e.g., drop of social life, loss of social activities, time constraints for social activities);  **Drop of personal care** (limited time for personal needs, non-existent time for personal needs);  **Changes in employment arrangements** (e.g., work pattern, shift to part time from full time, job loss);  **Family balance** (family time limits, family patterns change, limited family interaction);  **Professional/formal help** (e.g., formal help hiring, full-time help in the house);  **Time-management** (e.g., time constraints, time giving-up, lack of time for personal things). |
| Category  (Question) | Initial codes - Quotes | Refined codes | **Final themes** (e.g., refined codes used) |
| Rising action  (7. Can you describe what your average day looks like?) | Waking up early in the morning; providing help with medications; assisting with hygiene needs; dedicating all free time to the care recipient; early wake up; assisting with the showering and dressing; distributing medicines; day care center; going to work; loss of personal time; weekend dedicated to the care recipient; early morning wakeup; hygiene and medication help; day care center; afternoon organized around her; spouse tends to children; night terrors and lack of sleep for everyone; waking up at 4:30; working till 8:30am and returning to help with hygiene and food; day care; lunch; rest of the day completely dedicated to her; Saturday and Sunday dedicated to her; wake up 7:15; wash her and help her get dressed; day care center; house chores; putting her to sleep at 9; wake up at 7; assist with care neds; breakfast and medications; day dedicated to her; refuses to stay alone; wake up 7:30; self-care; breakfast and medication for him; grocery shopping; walking; lunch, quality time; bed time; wake up early; tend to the house; work time; caring for them in the afternoon till evening; sleep for a couple of hours at night; early wake up; house duties; his hygiene needs; medications; lunch time; nap; dinner time; professional caregiver present; 9pm bed time; early wake up; hygiene needs; breakfast preparation; mutual time; lunch time; dinner; bedtime; lack of sleep at night; | Early morning wake-up; Showering and dressing the care recipient; Medication organizing and delivery; Mealtime; Day care center preparation; Home stay; lunch time and cooking; afternoon time with care recipient/activities with care recipient; evening time and preparation for sleep/helping with preparation and going to bed). | **Early wake up** (e.g., early morning wake-up);  **Assistance with hygiene, food, and medicines** (e.g., showering and dressing the care recipient, medication organization and delivery, mealtime);  **Home care/Day care** (e.g., day care center preparation, home stay);  **Meals** (e.g., lunch time and cooking);  **Mutual quality time** (e.g., activities with care recipient, evening time);  **Bedtime** (e.g., preparation for sleep, helping with preparation, going to bed). |
| Category  (Question) | Initial codes - Quotes | Refined codes | **Final themes** (e.g., refined codes used) |
| Rising action  (8. What are some of the situations that you find most psychologically and emotionally challenging in your caregiving role?) | Loosing the main functions while still young; sitting on the couch and watching “through” TV; psychological breakdown; sense of abandonment/being alone in the decision to provide care; remaining patient; physical tiredness; psychological tiredness; emotional tiredness; not being able to spend time with children; sense of isolation; loss of friends; sense of guilt for not having the time for everyone; powerlessness towards the situation; sense of inadequacy; overwhelmed by the care responsibilities; not being heard; being ignored or left by those who have equal responsibility for care; the moments of aggression; no freedom of movement; loss of independence; being financially dependent on others for the caregiving expenses; being single/primary caregiver; seeing the weaknesses of the care recipient who use to be care taker; assessing/not assessing the emergency situations; feeling angry and lonely/abandoned; lack of communication with the care recipient; patience; becoming a mother of the parents; witnessing the suffering that is irreversible; feeling rejected; adapting to illness; feeling oppressed due to lack of freedom; the change of roles from daughter into being a mother of my mother; continuous agony of not being recognized by your parent; the inability to cure the disease; sense of powerlessness towards the disease; leaving her in nursing home; | Abandonment/being left alone in the care situation;  Loneliness; Being responsible for the health-related decisions; Fear of outcomes/ fear of decision related outcomes; personal time/freedom to plan personal time/spend personal time; Lack of training/knowledge; Feeling inadequate for the role/part of the role; Inability to change the course of illness/outcomes of illness; anger/negative perception of the role and events within the role; role-shift/not being able to be a “mother” to the parent. | **Fear** (e.g., fear of outcomes, fear of decision-related outcomes);  **Disappointment** (anger, negative perception of the role, negative perception of the events within the role, role-shift/not being able to be a “mother” to the parent);  **Powerlessness towards the irreversible change** (e.g., inability to change the course of illness, inability to change the outcomes of the illness);  **Loneliness** (e.g., abandonment/being left alone in the care situation, loneliness);  **Sense of inadequacy** (e.g., lack of training, lack of knowledge, feeling inadequate for the role, feeling inadequate for some parts of the role);  **Lack of personal freedom** (e.g., personal time, freedom to plan personal time, freedom to spend personal time). |
| Category  (Question) | Initial codes - Quotes | Refined codes | **Final themes** (e.g., refined codes used) |
| Climax/Critical point  (9. Can you describe the most critical points that occurred in your caregiving role and made it difficult to continue the caregiving role?) | Father’s denial for the care needs; inability to maintain work; difficulty focusing on anything but the care recipient; personal health issues; the provision of assistance becomes impossible; lack of family support; lack of involvement of other family members; care cannot be provided due to personal illness; choosing between care recipient and children; epileptic crisis where caregiver did not know what to do; personal health issues; having to force her to day care; missing personal life; feeling over as a person; seeing no perspective of the future; not knowing if the momentary health stat of the care recipient is serious or not; not knowing what the outcome of the short term colds will be; feeling guilty for keeping him in life while knowing he would refuse that if aware; the moment he became aware of his state and wished to be killed; progressive physical and emotional fatigue; inability to find professional assistance; combining work and care; witnessing her gradual decay; seeing the care recipient being abandoned by everyone else; physical fatigue that prevented me to see the situation clearly; days in which nothing else can be done; accepting his change; fear of confronting the fact that there is no going back; fact that my siblings refuse to share the responsibility for care; feeling alone and criticized by my siblings; being angry, frustrated and resentful; loneliness; | Fearing the outcomes; Negative emotional experiences/  tiredness/exhaustion;  Tired of advice or criticism; Physical exhaustion/health issues preventing care; feeling hopeless about the continuation of care that leads to no positive outcomes; fatigue/physical; fatigue/emotional; distrust in personal strength and ability to continue care provision; facing possible death/facing loss; inability to further contribute to care recipients care. | **Fear of loss/facing the possibility of loss/ facing the reality** (e.g., facing possible death**,** facing the idea of loss);  **Facing the fact that care cannot be given anymore** (e.g., distrust in personal strength and ability to continue care  **Psychological and emotional exhaustion** (e.g., negative emotional experiences, tiredness, exhaustion, tired of advice/criticism, physical exhaustion, health issues preventing care, feeling hopeless about the continuation of care that leads to no positive outcomes, fatigue/emotional). |
