# Supplementary material for: Using the Transformative Storytelling Technique to Generate Empowering Narratives for Informal Caregivers: Semistructured Interviews, Thematic Analysis, and Method Demonstration
Source: JMIR Form Res. 2022 Aug 2;6(8):e36405. doi: 10.2196/36405 (PMC9382549; doi:10.2196/36405)
Supplement: Multimedia Appendix 2 [file formative_v6i8e36405_app2.docx]

Caregiver Story

I remember that day as if it was yesterday, I was sitting in the doctor’s office. I was feeling so worried about her.

In fact, I have been worried about her for quite some time now.

She hasn’t been herself for over three months now. Although some of her friends told me that it was even longer than that. I was truly busy with my work, with my life. It seemed as if she didn’t really need me.

But now, all of the sudden here I was, in the doctor’s office, waiting for him to confirm my biggest fear.

“I am sorry I have to tell you this, but her condition is rapidly progressing. Is she staying alone home? She will need a lot of home care, someone to overview her daily life” – the doctor said.

We left the doctor’s office.

On our drive back home, she was quiet. She was looking through the window as if she wasn’t even there.

There were million thoughts running through my head, I had to get home to help my son with some tasks and make some dinner. I was wondering if there was any food in her fridge so that I could cook something for her before I head home.

“Tomorrow I will organize the rest and think about what doctor said”, I thought to myself.

“Are you driving me home?” She asked, suddenly breaking the silence.

“Yes” I responded.

“Good”, she said, “I can’t wait to see my father, I have so much to tell him about today”.

“Your father? What do you mean your father, your father died years ago”.

“No, he didn’t, no he didn’t you are lying”, she yelled in extreme distress. All this was taking place while I was driving.

“Take me home right now!” – she said. *[act her raising her voice slightly]*

“Okay, okay”, I said, “I am taking you home”.

My heart was racing, there is no “tomorrow”, I must think right now about what the doctor said. I absolutely cannot leave her alone tonight. This is how my story started.

I think I can describe it simply; I did not have a choice, but to take her with me that night**.** “It is my duty” – I said to myself - because she is my family. Besides, I cannot afford someone to take care of her.

As the days were going by, I had to change so many things in my life, one after another. I had never expected all of it to happen that way. I had to reorganize the space in my house and empty a room for her.

All my morning routine had to adapt to her from day one. I had to change my working hours and shift to part-time work because I couldn’t get to work by 9 any longer and stay there until 5. It is difficult sometimes to follow my own schedule.

My spouse often complains, and I do understand him, but I don’t see what else I can do. I can’t put her into a nursing home. My children didn’t seem bothered at the beginning. In fact, they were excited to have grandma home, but there comes a time when they try to make plans for us to go to the park on the weekend, as we use to, but I can’t promise anything because I do not know if she will be in her good mood that weekend and able to leave the house. My family balance is completely disturbed.

I remember my childhood weekends. Those times when I was being taken care of. I was so careless and happy. My mother was sometimes organizing lunch time with our family friends. There would be many children around. The fathers were barbequing, while my mother and her friends would bring the radio out and talk and laugh for hours. Those were some of the most beautiful memories I have from my childhood.

Before all this happened, I used to meet my friends at least two or three times a week for some wine or afternoon coffee. But now, I have almost lost touch with all my friends. They are calling, but I can never join them, and I think they are tired of calling me. There is always something happening, and I don’t have the time to go. I think they simply don’t understand me. They are not in my situation.

Every day is different, and you end up living with the illness yourself. You adapt to it and with time you try to learn how to handle it. My morning is starting at 5 now. I used to wake up at 7. But those days are now gone.

In the morning, I wake up and quickly prepare for the day so that I can wake her up and help her shower and get dressed. Sometimes she gets upset about the clothes I pick for her and refuses to wear them but if I let her choose alone, she picks summer clothes in the wintertime. All this can take around two hours.

Then we go to the kitchen together, and I pretend I need her help because that is the only way I can convince her to have breakfast. I ask her to put some marmalade on the bread for her, for me and for the kids when they wake up. She is busy with the marmalade while I am packing her lunch to take to the day care. In the meantime, I am waking up my kids for school.

Once we share a slice of marmalade bread, I give her the medications and walk with her outside where she is picked up by the day care centre bus. She stays at day care from 8 in the morning until 12 in the afternoon.

In the afternoon, after we all have had lunch together, the kids will go outside with their friends to play. And we stay home alone. On most of the days, when she feels fine, we talk about her memories about her parents. She likes to remember those days. It seems that this gives her some sort of comfort. And I like to see her happy.

We have some coffee and share occasional laughs. I smile when she smiles. There are moments when I feel that her “old self” is back, just the way she used to be. I feel this huge happiness in those moments. She asks me about the kids and how they are doing at school. I show her some of their homework, or recent photos with their friends. On other days she is completely disoriented and spends the time sitting quietly; it almost seems as If she is afraid. But then I talk to her about her parents, and she seems to calm down. Our evenings end with me putting her to bed and then doing the rest of the house chores before I go to bed.

One morning I woke up, and she was already awake, showering all by herself. On the bed she had prepared the clothes that she would wear that day. I can’t explain how I felt in that moment. That was after three years of care. Everything seemed to have finally fallen into place, “She is fine again”, I thought. My life could be fine again. Suddenly I felt so energetic. For a moment I started planning how we could reorganize our life and go back into the old routine. But soon, my happiness was gone. She came out from the bathroom and said she was late for work. We went downstairs and I didn’t know what to do. I took the marmalade out, and I was telling her that she needs to eat because they were coming to drive her to day care, and she became aggressive. She threw down the plate and yelled that she was going to work. My children heard everything, came into the kitchen to see what was happening. They were really scared. She didn’t recognize them, and I think she wasn’t recognizing me.

Fear, this is how I can describe such moments. I did not know what to do. I was scared for her, for my children. It was fear that didn’t let me think for a moment. I felt powerless because she wasn’t getting any better; she was either the same or getting worse. I couldn’t make her get better and I think I was making her worse by not knowing what to do exactly. I was completely alone. No one could truly understand what I was going through. I didn’t know what to do and I had no one to call. Everyone I knew had already advised me many times to consider nursing home. I couldn’t just take a break to have a moment for myself to think. It had been like that for months. I had lost my personal freedom. I felt trapped.

I took the phone and called ambulance.

She was taken to the hospital under extreme distress. She was aggressive, upset, and disoriented. I only felt fear.

In the ambulance car, she was restrained. It is painful to see your parent like that. I couldn’t handle her at home, and they couldn’t handle her in the ambulance. A thought crossed my mind “What if this is the last time I am seeing her?”

I was sitting in the hospital hallway, completely exhausted. It was psychological and emotional exhaustion*.* It seemed as if the world had disappeared. I wanted to take care of her, I wanted to do everything, but I seemed to be unable to even move from the chair I was sitting on. I was confronted with all my feelings and realized that I couldn’t continue like that anymore. I was praying that she was alive and well and I could take her back home. After a while the doctor came in and told me she is okay.

“But what should I do if she is not okay again? What shall I do when she becomes like this?” I asked the doctor.

He gave me a look of sympathy and said that there is only as much as a person can do before there is nothing else left to do.

It was the first time I had realized and accepted that I needed help. In the house, I could manage. But inside my mind, I needed to know what was happening to me. It all happened so fast and then it slowed down, it became like a new life I had not chosen. Now when I think of everything that happened during the last three years, it helps me see and understand how this part of my life unraveled.

It matters how you will tell your story*.* Sharing your story and organizing your thoughts is such a powerful way of changing the perspective on everything that happens to us.

Now I always pay attention how I think of the events inside of my mind, and how I verbalize them. I feel like I was not admitting how hard it was for me to start the caregiving, but also, I feel I was so tired and worn out that I overdramatized some events. I don’t know, this is how I see it now.

People don’t often think of giving the voice to their story. How would this story sound? Everything has a beginning, the moment where things slightly or suddenly changed, and you had to follow it by changing too. If I had known what was going to happen to me, I would have been able to get ready. I would have known better. Not only for the illness, but also mentally ready.

Now I am visiting self-help group in one association for caregivers. It’s free of charge.

I am learning how to tell my story to others, and I can see others feel better when they hear my story. But I also feel better because they truly understand me. They don’t feel sorry for me, they feel what I feel. One day I took a notebook, and I drew a line, and I started marking everything on this line, chronologically. I drew a circle on the line to symbolize the beginning, and under that I wrote how it started. Then the next circle was what happened after it all started, how it all had to change. Then I drew a circle for my daily routine, how my day looks like now, what I normally do. Also, I added a circle for the problems that arise, when I think I can’t do all the caregiving anymore.

This is my moment of the day when I have time to make and remake my story. On some days I can see what my problems were two weeks ago, and I laugh about them. Sometimes it’s better, sometimes it’s worse.

Once, in the self-help group, we were telling our story as if it was something that we heard from our friend. One different perspective, and I felt different about it too. There was a man who has just joined us, he listened carefully. He had just started taking care of his father, and it was obvious that it was all new to him.

We collect these stories now in the group. We were saying we have to make a book out of all our experiences. Anyone should make a small book out of his caregiving. I wish I had such a book when I started taking care of my mother, everything would be different. I think I would also be different.

It is important to know how to re-focus positively when all seems dark. How to accept things sometimes, instead of opposing life. I am learning this now.

I am taking a deep breath and hope for a fresh new day tomorrow.
